# Recent trends in antipsychotic polypharmacy in the treatment of schizophrenia

**DOI:** 10.1002/npr2.12127

**Published:** 2020-07-16

**Authors:** Norio Yasui‐Furukori, Kazutaka Shimoda

**Affiliations:** ^1^ Department of Psychiatry Dokkyo Medical University School of Medicine Mibu Japan

**Keywords:** antipsychotic polypharmacy, drug interactions, guidelines, Japan, schizophrenia

## Abstract

Ichihashi et al reported that 43% of patients had antipsychotic polypharmacy. Number of antipsychotics used in patients with schizophrenia in Japan was the greatest among Asian countries. However, the antipsychotic polypharmacy rate in Japan decreased gradually. Recent systematic review, meta‐analysis and meta‐regression analysis demonstrated that antipsychotic augmentation was superior to monotherapy. However, several cohort studies have suggested a significant association between antipsychotic daily dose and mortality. In addition, most pharmacokinetic interactions with antipsychotics occur at the metabolic level and usually involve changes in the activity of the major drug‐metabolizing enzymes involved in their biotransformation. Thus, avoidance of unnecessary polypharmacy, knowledge of the interaction profiles of individual agents, and careful individualization of dosage based on close evaluation of clinical response and possibly plasma drug concentrations are essential to prevent and minimize potentially adverse drug interactions in patients receiving antipsychotics.

Ichihashi et al^1^ reported that 43% of 1164 patients (43% in 23 university hospitals, 54% in 12 national/public hospitals, and 33% in 9 private hospitals) had antipsychotic polypharmacy in “Effectiveness of Guidelines for Dissemination and Education in psychiatric treatment (EGUIDE)” project in 2016, which is a series of training courses for Japanese psychiatrists.^2^

Antipsychotics are not always effective in schizophrenia patients. There are still patients with treatment‐resistant schizophrenia (TRS), which has led to a growing trend of resorting to atypical antipsychotic polypharmacy due to fewer side effects.^3^

Number of antipsychotics used and psychotropic drug loading in patients with schizophrenia in Japan was the greatest among Asian countries.^4^ In addition, Qiu et al^5^ reported the difference in antipsychotic polypharmacy trends between China and Japan. The overall antipsychotic polypharmacy rate was higher in Japan than in China. However, the antipsychotic polypharmacy rate in Japan decreased gradually, and in 2014, the rate in Japan was similar to that in China. Japanese governments published a press release to draw attention to the polypharmacy of psychotropic agents in 2011 and have regulated medical service fees in patients treated with antipsychotic polypharmacy since 2014, just after national reports in 2011 published an antipsychotic polypharmacy rate of 28.8%‐30.0% from 2005 to 2009.

A recent systematic review, meta‐analysis and meta‐regression analysis demonstrated that antipsychotic augmentation was superior to monotherapy regarding total symptom reduction, although the superiority was only apparent in open‐label and low‐quality trials and not in double‐blind and high‐quality trials.^6^ In addition, combining aripiprazole with clozapine was associated with the lowest risk of rehospitalization, indicating that certain types of polypharmacy may be feasible in the treatment of schizophrenia.^7^

On the other hand, several guidelines consistently recommend monotherapy of antipsychotic agents. For example, the Maudsley Prescribing Guidelines in Psychiatry states that we should concern ourselves with the addition of an antipsychotic to another antipsychotic solely to increase efficacy. From a theoretical point of view, since all antipsychotics block D2 receptors (unlike antihypertensives, which use different mechanisms), there is limited rationale for additional antipsychotics. In addition, many clinical trials studying the side effects of polypharmacy have associated polypharmacy with an increased incidence of side effects, although most have been uncontrolled studies or observational studies.^8^ Polypharmacy increases the risk of extrapyramidal syndrome and elevation of plasma prolactin concentration,^9^ except aripiprazole.^10^ Furthermore, anticholinergics were frequently used for patients who underwent polypharmacy.^11^

Several cohort studies have suggested a significant association between antipsychotic daily dose and mortality.^12^, ^13^ It is natural that daily doses of polypharmacy tend to be higher than that of monotherapy. In addition, most pharmacokinetic interactions with antipsychotics occur at the metabolic level and usually involve changes in the activity of the major drug‐metabolizing enzymes involved in their biotransformation, that is, the cytochrome P450 (CYP) monooxygenases and/or uridine diphosphate‐glucuronosyltransferases (UGT).^14^ Therefore, even if the dose of antipsychotics is low, the plasma concentration of each drug may be high after polypharmacy, leading to a risk of mortality.

Mortality associated with antipsychotic polypharmacy has not yet been concluded. Previous reports have suggested that significant mortality risks are 2.46 (95% CI, 1.10‐5.47)^15^ and 2.50 (95% CI, 1.46‐4.30).^16^ However, later studies have reported no association between mortality and antipsychotic polypharmacy.^17^ Tiihonen et al demonstrated that compared with antipsychotic monotherapy, concomitant use of 2 or more antipsychotics was not associated with increased mortality (HR, 0.86; 95% CI, 0.51‐1.44).^17^ Although the principal outcome measure, time to all‐cause discontinuation, indicated superiority for monotherapy over polypharmacy for the majority of (oral and depot) second‐generation APs (SGAs), a significant overall advantage of polypharmacy was detected for mortality and hospitalization.^18^ The risk of natural death did not increase with the number of concurrently used antipsychotic agents compared with the corresponding risk associated with antipsychotic monotherapy (adjusted odds ratio [OR] = 1.48 [95% CI, 0.89‐2.46]; 2 antipsychotics: OR = 0.91 [95% CI, 0.61‐1.36]; 3 or more antipsychotics: OR = 1.16 [95% CI, 0.68‐2.00]).^19^

Thus, although evident disadvantage of antipsychotic polypharmacy has not yet been concluded, avoidance of unnecessary polypharmacy, knowledge of the interaction profiles of individual agents, and careful individualization of dosage based on close evaluation of clinical response and possibly plasma drug concentrations are essential to prevent and minimize potentially adverse drug interactions in patients receiving antipsychotics.

## CONFLICT OF INTEREST

Norio Yasui‐Furukori has been a speaker for Otsuka Pharmaceutical Co., Ltd., Mochida Pharmaceutical Co., Ltd., Dainippon‐Sumitomo Pharmaceutical Co., and MSD Co. Kazutaka Shimoda has received research support from Novartis Pharma KK, Dainippon Sumitomo Pharma Co., Astellas Pharma Inc, Meiji Seika Pharma Co., Ltd., Eisai Co., Ltd., Pfizer Inc, Otsuka Pharmaceutical Co., Ltd., Daiichi Sankyo Co., and Takeda Pharmaceutical Co., Ltd., and honoraria from Eisai Co., Ltd., Mitsubishi Tanabe Pharma Corporation, Takeda Pharmaceutical Co., Ltd., Meiji Seika Pharma Co., Ltd., Janssen Pharmaceutical KK, Shionogi & Co., Ltd., Dainippon Sumitomo Pharma Co., Daiichi Sankyo Co., and Pfizer Inc The funders did not have any role in data collection or in the study design, analysis, decision to publish, or preparation of the manuscript. The remaining authors declare that they have no competing interests to report.

## AUTHOR CONTRIBUTION

NYF designed the study and wrote the initial draft of the manuscript. KS contributed to supervision of the draft.

## Data Availability

Data sharing is not applicable to this article as no datasets were generated or analyzed during the current study.

## References

[1] Ichiahshi K , Hori H , Hasegawa N , Yasuda Y , Yamamoto T , Tsuboi T , et al. Prescription patterns in patients with schizophrenia in Japan: first‐quality indicator data from the survey of “Effectiveness of Guidelines for Dissemination and Education in psychiatric treatment (EGUIDE)’’ project. Neuropsychopharmacology Reports. 10.1002/npr2.12122 (in press). PMC7722678 32602667

[2] Takaesu Y , Watanabe K , Numata S , Iwata M , Kudo N , Oishi S , et al. Improvement of psychiatrists' clinical knowledge of the treatment guidelines for schizophrenia and major depressive disorders using the 'Effectiveness of Guidelines for Dissemination and Education in Psychiatric Treatment (EGUIDE)' project: A nationwide dissemination, education, and evaluation study. Psychiatry Clin Neurosci. 2019;73(10):642–8. 31437336 10.1111/pcn.12911 PMC6852015

[3] Mojtabai R , Olfson M . National trends in psychotropic medication polypharmacy in office‐based psychiatry. Arch Gen Psychiatry. 2010;67(1):26–36. 20048220 10.1001/archgenpsychiatry.2009.175

[4] Yang S‐Y , Chen L‐Y , Najoan E , Kallivayalil RA , Viboonma K , Jamaluddin R , et al. Polypharmacy and psychotropic drug loading in patients with schizophrenia in Asian countries: Fourth survey of Research on Asian Prescription Patterns on antipsychotics. Psychiatry Clin Neurosci. 2018;72(8):572–9. 29761577 10.1111/pcn.12676

[5] Qiu H , He Y , Zhang Y , He M , Liu J , Chi R , et al. Antipsychotic polypharmacy in the treatment of schizophrenia in China and Japan. Aust N Z J Psychiatry. 2018;52(12):1202–12. 30309245 10.1177/0004867418805559

[6] Galling B , Roldán A , Hagi K , Rietschel L , Walyzada F , Zheng W , et al. Antipsychotic augmentation vs. monotherapy in schizophrenia: systematic review, meta‐analysis and meta‐regression analysis. World Psychiatry. 2017;16(1):77–89. 28127934 10.1002/wps.20387 PMC5269492

[7] Tiihonen J , Taipale H , Mehtälä J , Vattulainen P , Correll CU , Tanskanen A . Association of antipsychotic polypharmacy vs monotherapy with psychiatric rehospitalization among adults with schizophrenia. JAMA Psychiatry. 2019;76(5):499–507. 30785608 10.1001/jamapsychiatry.2018.4320 PMC6495354

[8] Misawa F , Shimizu K , Fujii Y , Miyata R , Koshiishi F , Kobayashi M , et al. Is antipsychotic polypharmacy associated with metabolic syndrome even after adjustment for lifestyle effects? A cross‐sectional study. BMC Psychiatry. 2011;11:118. 21791046 10.1186/1471-244X-11-118 PMC3155482

[9] Gallego JA , Nielsen J , De Hert M , Kane JM , Correll CU . Safety and tolerability of antipsychotic polypharmacy. Expert Opin Drug Saf. 2012;11(4):527–42. 22563628 10.1517/14740338.2012.683523 PMC3384511

[10] Yasui‐Furukori N , Furukori H , Sugawara N , Fujii A , Kaneko S . Dose‐dependent effects of adjunctive treatment with aripiprazole on hyperprolactinemia induced by risperidone in female patients with schizophrenia. J Clin Psychopharmacol. 2010;30(5):596–9. 20814333 10.1097/JCP.0b013e3181ee832d

[11] Chakos MH , Glick ID , Miller AL , Hamner MB , Miller DD , Patel JK , et al. Baseline use of concomitant psychotropic medications to treat schizophrenia in the CATIE trial. Psychiatr Serv. 2006;57(8):1094–101. 16870959 10.1176/ps.2006.57.8.1094

[12] Cullen BA , McGinty EE , Zhang Y , dosReis SC , Steinwachs DM , Guallar E , et al. Guideline‐concordant antipsychotic use and mortality in schizophrenia. Schizophr Bull. 2013;39(5):1159–68. 23112292 10.1093/schbul/sbs097 PMC3756776

[13] Torniainen M , Mittendorfer‐Rutz E , Tanskanen A , Bjorkenstam C , Suvisaari J , Alexanderson K , et al. Antipsychotic treatment and mortality in schizophrenia. Schizophr Bull. 2015;41(3):656–63. 25422511 10.1093/schbul/sbu164 PMC4393693

[14] Spina E , de Leon J . Metabolic drug interactions with newer antipsychotics: a comparative review. Basic Clin Pharmacol Toxicol. 2007;100(1):4–22. 17214606 10.1111/j.1742-7843.2007.00017.x

[15] Waddington JL , Youssef HA , Kinsella A . Mortality in schizophrenia. Antipsychotic polypharmacy and absence of adjunctive anticholinergics over the course of a 10‐year prospective study. Br J Psychiatry. 1998;173:325–9. 9926037 10.1192/bjp.173.4.325

[16] Joukamaa M , Heliövaara M , Knekt P , Aromaa A , Raitasalo R , Lehtinen V . Schizophrenia, neuroleptic medication and mortality. Br J Psychiatry. 2006;188:122–7. 16449697 10.1192/bjp.188.2.122

[17] Tiihonen J , Suokas JT , Suvisaari JM , Haukka J , Korhonen P . Polypharmacy with antipsychotics, antidepressants, or benzodiazepines and mortality in schizophrenia. Arch Gen Psychiatry. 2012;69(5):476–83. 22566579 10.1001/archgenpsychiatry.2011.1532

[18] Katona L , Czobor P , Bitter I . Real‐world effectiveness of antipsychotic monotherapy vs. polypharmacy in schizophrenia: to switch or to combine? A nationwide study in Hungary. Schizophr Res. 2014;152(1):246–54. 24275583 10.1016/j.schres.2013.10.034

[19] Ray WA , Chung CP , Murray KT , Hall K , Stein CM . Atypical antipsychotic drugs and the risk of sudden cardiac death. N Engl J Med. 2009;360(3):225–35. 19144938 10.1056/NEJMoa0806994 PMC2713724

